# Review of Novel Potential Insulin Resistance Biomarkers in PCOS Patients—The Debate Is Still Open

**DOI:** 10.3390/ijerph19042099

**Published:** 2022-02-13

**Authors:** Jagoda Kruszewska, Hanna Laudy-Wiaderny, Michał Kunicki

**Affiliations:** 1 Laboratory of Centre for Preclinical Research, Department of Experimental and Clinical Physiology, Medical University of Warsaw, 02-097 Warsaw, Poland; 2 Independent Public Healthcare Complex in Masovian Minsk, 05-300 Masovian Minsk, Poland; hlaudywiaderny@gmail.com; 3 Department of Gynecological Endocrinology, Medical University of Warsaw, 00-315 Warsaw, Poland; mkunicki@op.pl; 4 INVICTA Fertility and Reproductive Center, 00-019 Warsaw, Poland

**Keywords:** polycystic ovarian syndrome, insulin resistance, galectin, neuregulin, preptin, myonectin, gremlin, omentin, nesfatin, xenin

## Abstract

Research on proteins and peptides that play roles in metabolic regulation, which may be considered potential insulin resistance markers in some medical conditions, such as diabetes mellitus, obesity and polycystic ovarian syndrome (PCOS), has recently gained in interest. PCOS is a common endocrine disorder associated with hyperandrogenemia and failure of ovulation, which is often accompanied by metabolic abnormalities, including obesity, dyslipidemia, hyperinsulinemia, and insulin resistance. In this review, we focus on less commonly known peptides/proteins and investigate their role as potential biomarkers for insulin resistance in females affected by PCOS. We summarize studies comparing the serum fasting concentration of particular agents in PCOS individuals and healthy controls. Based on our analysis, we propose that, in the majority of studies, the levels of nesfastin-1, myonectin, omentin, neudesin were decreased in PCOS patients, while the levels of the other considered agents (e.g., preptin, gremlin-1, neuregulin-4, xenopsin-related peptide, xenin-25, and galectin-3) were increased. However, there also exist studies presenting contrary results; in particular, most data existing for lipocalin-2 are inconsistent. Therefore, further research is required to confirm those hypotheses, as well as to elucidate the involvement of these factors in PCOS-related metabolic complications.

## 1. Introduction

Due to the increasing worldwide prevalence of obesity and its undeniable relationship with decreased insulin sensitivity of the peripheral cells, there has been a growing interest in the study of proteins/peptides that may regulate metabolic homeostasis [1,2,3,4]. To date, most research has been devoted to factors deriving directly from the white adipose tissue (WAT)—also known as adipokines—including adiponectin, resistin, leptin, visfatin, apelin, retinol-binding protein 4, and chemerin, to name but a few [5,6,7,8,9]. It has been shown that adipose tissue may function as an endocrine organ and that it secretes several adipokines, thus constituting a link between body mass excess and glucose level disturbances [7,10]. Adipokines may be synthesized in excess, or their expression may be diminished, in several conditions associated with insulin resistance, such as obesity, type 2 diabetes mellitus (T2DM), gestational diabetes mellitus (GDM), or polycystic ovarian syndrome (PCOS) [5,8]. 

PCOS is a frequent endocrine disorder associated with hyperinsulinemia and hyperandrogenemia, which is often accompanied by infertility and obesity [11]. Affected individuals are at increased risk of developing cardiovascular and metabolic diseases, as well as anxiety disorders [12].

The condition is most commonly diagnosed according to the Rotterdam criteria, when two out of the three following features occur (after the exclusion of related disorders): oligo/anovulation, clinical and/or biochemical hyperandrogenism, or polycystic ovaries on ultrasound [13,14]. Beside the Rotterdam criteria proposed by the European Society for Human Reproduction and Embryology/American Society for Reproductive Medicine (ESHRE/ASRM) [13,14], there exist other societies, such as National Institutes of Health/National Institute of Child Health and Human Disease (NIH/NICHD) and the Androgen Excess and PCOS Society, which have presented slightly different criteria for the recognition of PCOS [12].

The prevalence of PCOS has been estimated as 4–26% depending on the studied population (in terms of age, ethnicity, and so on) and applied criteria. It has been shown that adoption of the NIH/NICD guidelines may account for recognition of PCOS in 4–8% adult female patients, while this rate may be as high as 15–20%, when ESHRE recommendations are acknowledged [12].

Nevertheless, despite the high prevalence of the disorder, we still know little about its exact pathogenesis. Over the years, many hypotheses have been introduced, including genetic, epigenetic, and environmental factors [15]. The key pathogenetic factors include hyperandrogenemia, subclinical inflammation, and defective insulin signaling [16]. It has been evidenced that up to 50–80% of women with PCOS are diagnosed with insulin resistance [10,12,17]. Hyperinsulinemia is responsible for metabolic and cardiovascular complications [18], as well as decreasing sex hormone binding globulin (SHBG) production in the liver (resulting in an increase of free and bioactive androgen levels in the circulation) and potentiating the luteinizing hormone (LH)-dependent effect on the ovarian cells, leading to the enhanced synthesis of androgens [5]. Hyperandrogenemia in females further aggravates the course of metabolic complications [19,20]. 

Recent studies have also focused on the dysregulation of intestinal microflora in PCOS individuals [21,22,23] and abnormalities in the level of metabolites generated by bacteria, such as bile acids (BAs), short-chain fatty acids (SCFAs), branched-chain amino acids (BCAAs), ceramides, and trimethylamine N-oxide (TMAO) [17]. Interestingly, it has been shown that an imbalance in the gut microbiota in women with PCOS may be conducive to dysfunction of the immune system, development of chronic low-grade inflammation, and enhancement of the synthesis of proinflammatory cytokines, all of which may lead to defective signaling through insulin receptors [21,23]. Therefore, we now know that gut dysbiosis may induce insulin resistance.

Moreover, it has been shown that the presence of some bacterial species may lead to altered secretion of several peptides involved in the metabolic homeostasis and appetite regulation; for example, an increase of Bacteroides species has been shown to be associated with the dysregulation of ghrelin and peptide YY [23].

Furthermore, there is also growing scientific interest in the study of microRNA (miRNA), which regulates gene expression at the post-transcriptional level and has been also shown to be altered in PCOS individuals [17,24].

Last, but not least, in patients with PCOS, many studies assessing the levels of adipokines have been conducted [5] and it has been indicated that the dysregulation of such hormones leads to insulin resistance [6].

At present, there are emerging data regarding peptides/glycoproteins, synthesized in other tissues than WAT, which may also be involved in the pathomechanism of insulin resistance. Some of them have been investigated as potential insulin resistance biomarkers in PCOS [5]. Studying new possible markers may not only provide the insight into the intricate pathomechanism of PCOS, but also implicates that measurement of several target proteins that may provide useful information about the severity of the condition, possible complications, and prognoses, or which may serve as helpful tool for the early detection of metabolic complications. The analysis of such markers may also play a significant role in monitoring of the course of a disease [15]. 

In this review, we present several novel proteins/peptides which may be considered as insulin resistance markers, and summarize their potential roles in the pathogenesis of PCOS. We focus on less-commonly known markers that have not been extensively reviewed: nesfatin, preptin, myonectin, omentin, gremlin-1, galectin-3, neuregulin-4, xenopsin-related peptide, xenin-25, neudesin, and lipocalin-2. Therefore, the aims of this review are to provide baseline information about these markers and their relation to insulin resistance, as well as discussing studies assessing the concentration of these markers in PCOS patients. 

We also investigated whether the observed variations in the serum concentrations of the markers are only the result of insulin resistance/excess body mass, or if there is evidence that they may affect the ovarian function in another, more direct manner.

## 2. Materials and Methods

This article is intended as a narrative review, updating and summarizing current knowledge on the most recent insulin resistance markers in women with PCOS. In order to identify these potential markers, we searched PubMed using phrases: ‘PCOS and insulin resistance’, ‘PCOS and metabolic syndrome’, and ‘PCOS and markers’. We included agents that, in the last decade, have been associated with insulin resistance in the course of PCOS and, in our opinion, no recent comprehensive review in terms of their relation to PCOS has been published. We qualified selected markers including nesfatin, preptin, myonectin, omentin, gremlin-1, galectin-3, neuregulin-4, xenopsin-related peptide, xenin-25, neudesin, and lipocaline-2.

We included publications restricted to the English language, including case-control trials, observational studies, review articles, and meta-analyses. The year of publication was not a limiting factor. The search was conducted up until November 2021.

Table 1 presents detailed information regarding the most recent study for each of the markers comparing serum fasting marker levels between PCOS women and healthy subjects. Thereby, we included studies in which the obtained results were mostly in agreement with the previous research (i.e., those which presented the most predominant tendency, in terms of the level of the marker). Moreover, these studies also provided information about the administered kit, established diagnosis basing on the Rotterdam criteria [13].

## 3. Nesfatin-1

**Figure 1:**
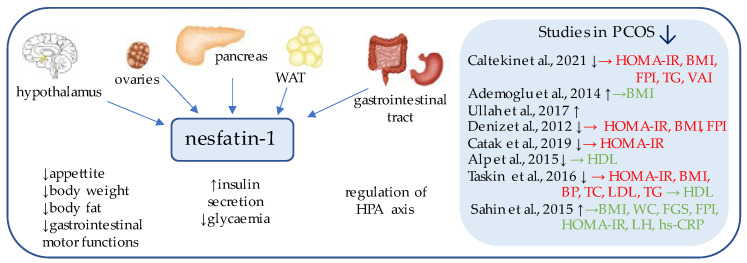
Role of nesfatin-1 in the pathogenesis of insulin resistance and PCOS. List of the studies assessing serum nesfatin level in the PCOS patients. ↑/↓ indicates whether concentration level of serum nesfatin was increased/decreased in PCOS individuals (*p* < 0.05); → (green)—indicates positive correlation between serum nesfatin and particular indicators; → (red)—indicates negative correlation between serum nesfatin and particular indicator; HOMA-IR—Homeostatic Model Assessment for Insulin Resistance; BMI—body mass index; FPI—fasting plasma insulin; TG—triglycerides; VAI—Visceral Adiposity Index; HDL—high density lipoprotein; BP—blood pressure; TC—total cholesterol; LDL—low-density lipoproteins; WC—waist circumference; FGS—Ferriman–Gallwey Score; LH—Luteinizing Hormone; hs-CRP—high-sensitivity C-Reactive Protein; WAT—white adipose tissue; HPA axis—Hypothalamic–Pituitary–Adrenal axis.

Nesfatin-1 (Figure 1) is an 82-amino acid neuropeptide derived from the post-translational processing of the N-terminal fragment of nucleobindin2 (NUCB2) [36], which was originally identified as an anorexigenic hypothalamic neuropeptide, a chronic intracerebroventricular injection of which reduced the food intake and decreased body weight and the amount of body fat [37]. Its expression has been detected in the areas responsible for appetite regulation, such as the arcuate, paraventricular, and supraoptic nuclei, as well as in the lateral hypothalamic area and zona incerta [37]. 

Subsequent studies have focused on the peripheral expression of nesfatin and proved its synthesis on mRNA and protein levels within the gut, pancreas, and white adipose tissue [37,38]. 

Interestingly, in the gastrointestinal tract, nesfatin-1 has been shown to be co-expressed in about 86% of x/A cells with the commonly known appetite-regulator ghrelin, and to a lesser extent, within the D cells with somatostatin and histamine-synthesizing enzyme histidine decarboxylase (HDC) [39,40]. Moreover, in the pancreas of rodents, beta cells co-localize insulin and pronesfatin immunoreactivity [41]. In animal studies, the peptide has been shown to exert antihyperglycemic effect [42]. Nesfatin also enhanced glucose-induced insulin secretion by promoting Ca2+ influx through L-type channels in mouse islet β-cells [43]. Taken together, nesfatin-1 can be described as an important energy homeostasis regulator. 

Furthermore, there are also hypotheses that nesfastin-1 may affect the function of the reproductive system [44]. In the central nervous system, nesfatin is also co-expressed in the same areas as other factors such as neuropeptide Y (NPY) and proopiomelanocortin (POMC), which affect gonadotropin-releasing hormone (GnRH) pulsations, prompting some scientists to conclude that nesfatin may centrally regulate the hypothalamic–pituitary–gonadal (HPG) axis and can have an impact on pituitary hormonal secretion [44]. Interestingly, intracerebroventricular administration of nesfatin increased luteinizing hormone (LH) secretion in pubertal, but not adult, rats; hence, it is possible that nesfatin may also be involved in the induction of puberty [45]. 

Interplay between the dysregulation of metabolism and the HPG axis is a characteristic feature of PCOS; therefore, nesfatin-1 analysis among such individuals may provide valuable data. In fact, several studies in letrozole or dihydrotestosterone (DHT)-induced PCOS rat models have confirmed this hypothesis, showing decreased concentrations of nesfatin-1 in the follicular fluid, as well as in the blood [46,47].

Similar studies have also been conducted on humans [25,38,48,49,50,51,52,53]. In most of them, PCOS population was characterized by decreased circulating nesfatin-1 concentration [25,49,50,51,52]; nevertheless, studies by Ademoglu, Sahin, and Ullah et al. showed the opposite, as serum nesfastin was increased [38,48,53]. Similar inconsistent data exist for type 2 diabetes mellitus (T2DM) [54,55]. In the studies by Deniz, Taskin, and Caltekin et al., negative correlations between nesfatin-1 levels and Body Mass Index (BMI) and Homeostatic Model Assessment-Insulin Resistance (HOMA-IR) have been observed [25,49,52]. 

The largest study has been provided by Taskin et al., who additionally subdivided PCOS subjects (*n* = 60) into two groups depending on their BMI (obese, *n* = 28, non-obese; *n* = 32). Interestingly, nesfastin-1 levels were significantly decreased in both obese and lean PCOS patients, in comparison to healthy age-matched controls; moreover, obese PCOS individuals had even lower serum nesfastin than their lean counterparts, where this difference was also significant [52]. These data suggest that nesfatin-1 may play a role in the pathogenesis of PCOS, as well as in obesity and insulin resistance.

## 4. Preptin

**Figure 2:**
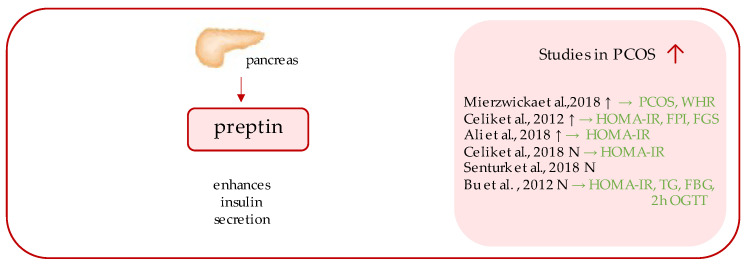
Role of preptin in the pathogenesis of insulin resistance and PCOS. List of the studies assessing serum nesfatin level in the PCOS patients. ↑/N indicates whether concentration level of serum preptin was increased/unchanged in PCOS individuals (*p* < 0.05); → (green)—indicates positive correlation between serum nesfatin and particular indicators; PCOS—polycystic ovarian syndrome; WHR—waist-to-hip ratio; HOMA-IR—Homeostatic Model Assessment for Insulin Resistance; FPI—fasting plasma insulin; FGS—Ferriman–Gallwey score; TG—triglycerides; FBG—fasting blood glucose; OGTT—oral glucose tolerance test.

Preptin (Figure 2) is a 34-amino acid peptide, a product of the same gene that encodes Insulin-like growth factor 2 (IGF II); more precisely, it corresponds to the Asp^69^–Leu^102^ sequence of the E-domain of its precursor, proinsulin-like growth factor II [56,57]. The peptide originated from the study of Buchanan et al., who purified it from secretory granules of cultured murine bTC6-F7 pancreatic b-cells [56], where preptin is co-secreted all together with insulin, amylin, and pancreastatin, and exhibits biological function as a glucose-mediated insulin secretion enhancer [1]. 

Over the years, several studies aimed at measuring fasting preptin serum concentrations in females with PCOS were undertaken; however, the results of which remain inconsistent, as significantly higher serum preptin concentrations have been detected in some studies [26,58,59], while other studies have undermined this relationship [60,61,62].

Initially, Celik et al. demonstrated significantly increased preptin levels in 25 females with PCOS, compared to their age- and BMI-matched controls [58]. Preptin was positively correlated with HOMA-IR and fasting plasma insulin (FPI), as well as Ferriman–Gallwey (FG) score. Later on, in a different study, Bu et al. divided both control and PCOS groups in two independent sub-groups, based on the glucose tolerance status of individuals and concluded that women with impaired glucose tolerance (IGT) had higher serum preptin levels, regardless of PCOS status, when compared with women with normal glucose tolerance (NGT). They also observed correlation with HOMA-IR [62]. Consequently, Senturk et al. also addressed the issue, but created sub-groups depending on individuals BMI values. Their analysis did not provide any significant correlations between PCOS patients and BMI-matched controls, as well as between each subgroup (overweight PCOS, normal weight PCOS, overweight control, and normal weight control) [61]. There have been no other studies in individuals with PCOS supporting the correlation between preptin and BMI [26,58,61]. Importantly, initial groups of PCOS and controls did not differ in terms of HOMA-IR between each other; hence, there is a possibility their study lacking statistical significance [61]. Therefore, in the study by Ali et al., while the PCOS vs. control groups differed significantly in terms of HOMA-IR, elevated circulating preptin concentrations were observed, which were positively correlated with insulin resistance markers [59]. Such a correlation was also reached in another study by Celik et al. [60].

Mierzwicka et al. have recently provided an observation on the largest cohort included in the review, and demonstrating significantly increased serum preptin in PCOS individuals (data presented in the table), consistent with the first Celik et al. study. Nevertheless, in both studies, the initial groups (PCOS vs. control) differed significantly in terms of HOMA-IR [26]; however, surprisingly and contrarily, the serum preptin was not correlated with insulin resistance markers, leading to the conclusion that elevated serum preptin might be shown to be an independent predictor of PCOS in the future, which stays in the contrary with former studies [26].

The data appear to be contradictory; hence, the status of preptin in the pathogenesis of PCOS remains not well-established. There seems to be a lot of emerging data confirming its link to insulin resistance, which is frequent in PCOS individuals. Similarly, its concentration has been shown to be increased in other disorders associated with glucose tolerance impairment, such as T2DM [63] or gestational diabetes mellitus (GDM) [64]. Thus, preptin may be acknowledged as a potential insulin resistance marker. However, its contribution to the pathogenesis of PCOS requires further research. We agree that it would be of value in future research to assess peptide concentrations after 2h-OGTT [26], as it is also believed that preptin may rather not initiate the insulin efflux but, instead, increases the second phase of glucose mediated secretion (by 30% in the animal study by Buchanan et al.) [56]. 

## 5. Myonectin

**Figure 3:**
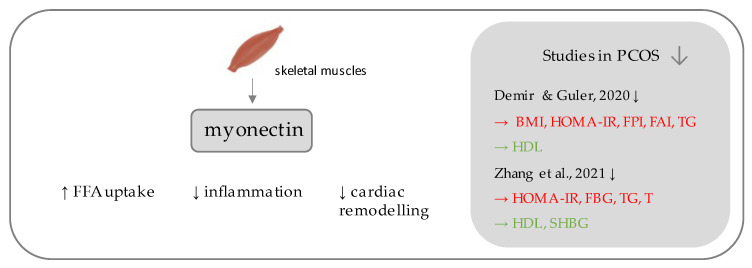
Role of myonectin in the pathogenesis of insulin resistance and PCOS. List of the studies assessing serum nesfatin level in the PCOS patients. ↓ indicates that concentration level of serum myonectin was decreased in PCOS individuals (*p* < 0.05); → (green)—indicates positive correlation between serum myonectin and particular indicators; → (red)—indicates negative correlation between serum myonectin and particular indicator; BMI—Body Mass Index; HOMA-IR—Homeostatic Model Assessment for Insulin Resistance; FPI—fasting plasma insulin; FAI—free androgen index; TG—triglycerides; HDL—high density lipoproteins; FBG—fasting blood glucose; T—testosterone; SHBG—sex hormone binding globulin.

Myonectin (Figure 3) belongs to the C1q/TNF-related proteins (CTRPs), constituting its most recent member, assigned as CTRP-15 [65,66]. CTRPs, due to presence of a C-terminal globular domain, with sequence homology to the immune complement protein C1q are further counted among the C1q protein family [66], together with a commonly known adipokine, adiponectin, which is a well-established insulin-sensitizing hormone [67], the diminished levels of which reflect insulin resistance, which has been repeatedly demonstrated in PCOS individuals [68,69]. Unlike the other CTRPs, which are widely expressed in many tissues, most predominantly in adipocytes [66], myonectin is an example of a myokine, with its highest expression occurring within the skeletal muscles [65]. Initially, its function was linked to the promotion of fatty acid uptake by adipocytes and hepatocytes, consequently leading to a reduction of serum free fatty levels [65]. Importantly, it has been shown to be up-regulated after feeding and physical exercise [65]. In the study by Little et al., when myonectin-deficient mice were administered a high-fat diet (HFD), they exhibited features associated with insulin resistance, had more elevated post-prandial very low-density lipoprotein (VLDL) and triglycerides (TG) levels, significantly increased fat mass (both visceral and subcutaneous fat depots), and, surprisingly, decreased liver steatosis, in comparison to control wild type (WT) litter-mates which were also administered HFD [70]. It may be concluded that myonectin plays a significant role in glucose and lipid metabolism and may constitute a link between skeletal muscle and the function of other organs [65]. Moreover, there are emerging data about positive pleiotropic effects of myonectin (such as inflammation suppression, improvement of endothelial function, protection against ischemia-reperfusion injury, and exertion of antifibrotic effect on cardiac remodeling) [71,72,73,74].

There have been two studies assessing the levels of myonectin in PCOS patients, to the best of our knowledge [27,75]. In the study by Demur and Guler, myonectin levels were significantly lower in PCOS subjects, when compared to their BMI-matched controls. Myonectin has exhibited inverse association with BMI, insulin resistance (measured using HOMA-IR, FPI), free androgen index (FAI), and triglycerides, while it showed a positive association with high-density lipoprotein cholesterol (HDL-C) [27]. Concordant results, in terms of myonectin concentration and the correlations were obtained by Zhang et al., in slightly larger groups (each containing 100 participants) [75]. The first aforementioned study also compared overweight/obese PCOS subjects (*n* = 40) to lean PCOS subjects (*n* = 32), and concluded that body mass excess may decrease myonectin concentration (6.34 ± 1.93 vs. 7.31 ± 1.90 ng/mL; *p = 0*.038) [27]. Similar observations have been made in the cohorts with obesity and T2DM, wherein serum myonectin was negatively correlated with HOMA-IR [76]. Importantly, the implementation of physical exercise, enhancement of insulin sensitivity, and body mass reduction may increase the myonectin serum levels [77]. The aforementioned studies remain promising, indicating that myonectin may be an important regulator of metabolism and that its concentration is decreased under conditions associated with insulin resistance and body mass excess, including PCOS.

There is also some discrepancy that has arisen in the scientific terminology, which may require rectification. Although myonectin was initially described in 2012, by Seldin et al., as a lipid turnover regulator [65], the same substance was referred to as an erythroferrone two years later and was said to function as an erythrocyte-derived regulator of iron metabolism, as the inhibitor of hepcidin [78]. These terms are often used interchangeably in the literature, depending on the main area of interest in the particular study [79]. Little is known about a link between these two apparently different types of mechanisms. Lawen proposed that iron uptake may be required not only for hemoglobin, but also for myoglobin production in muscles [80]. The pattern of activity may depend on the type of stimulus (e.g., feeding/exercise vs. erythropoietic stress/hemorrhage) [80], as when myonectin-deficient mice were administered a high fat diet (HFD), they did not present deviations in morphology [70]. Better understanding of the link between these two mechanisms may require further studies and the correlation between insulin resistance and iron metabolism may be considered in their design [79].

## 6. Omentin-1

**Figure 4:**
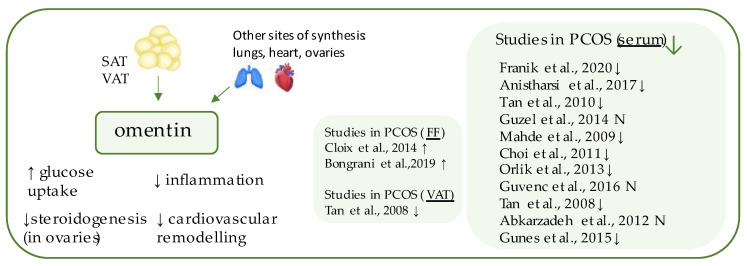
Role of omentin in the pathogenesis of insulin resistance and PCOS. List of the studies assessing nesfatin level in serum, follicular fluid (FF), and visceral adipose tissue (VAT) of the patients with PCOS. ↓/N indicates that concentration level of omentin was decreased/unchanged in PCOS individuals (*p* < 0.05).

Omentin-1 (Figure 4) is a 296-amino acid glycoprotein, a major form of the circulating omentins in human plasma, products of the gene localized within the 1q22–q23 chromosomal region which has been linked to T2DM in various populations [9,81]. It belongs to the adipokines, the expression of which occurs largely in the visceral (omental) and, to a twenty times lesser extent, in the subcutaneous adipose tissue and is a factor detectable in human serum [82,83]. Its identification originated in the study of Yang et al., who found it in abundance in the visceral fat complementary DNA (cDNA) library and observed that its synthesis mainly occurs in the stromal vascular cells, not the adipocytes themselves [82]. Previously, the presence of a protein with the same sequence (but in significantly smaller concentrations) was also detected in the intestine and endothelium; therefore, omentin may be found in the literature under other names, such as intelectin, endothelial lectin HL-1 (27a), and intestinal lactoferrin receptor [9,82,84]. Indeed, its function relies on increasing insulin-mediated glucose uptake by activating the protein kinase B (PKB or Akt) pathway, and decreased serum concentrations have been detected in individuals with obesity and T2DM [82,85]. It is predominantly positively related to adiponectin and negatively to BMI, leptin, and insulin resistance [85]. Therapies based on body weight reduction have been shown to contribute to elevation of its serum concentration [86,87]. Beside its anti-diabetic properties, omentin also decreases cardiovascular risk and preserves anti-inflammatory and antiatherogenic functions, through vasodilation of blood vessels, attenuation of C-reactive protein-induced angiogenesis, and reduction of tumor necrosis factor-α (TNFα)-induced inflammation in the endothelium and vascular smooth cells [88,89,90]. 

Studies assessing circulating omentin concentrations have been conducted among individuals with polycystic ovarian syndrome [28,81,86,91,92,93,94,95,96,97,98], where the majority of them ascertained diminished serum omentin levels [86,92,93,94,96,98], which have also been confirmed by the recent meta-analysis performed by Tang et al. [99]. The study by Tan et al. also confirmed lower omentin level in the visceral tissue of the patients with PCOS [96]. However, the data regarding, whether decreased serum omentin may be a result of PCOS, regardless of nutritional status, remain inconsistent. In some studies, a decrease of the omentin serum level could be explained by a higher incidence of obesity in the PCOS group, in comparison to the control subjects. Several case-control studies, in which initial groups (PCOS vs. control) were later sub-divided depending on BMI (normal weight vs. obese), are contradictory, as the data indicated no significant decrease in plasma adipokine in non-obese PCOS individuals [97]. To the contrary, other authors have concluded that PCOS is an independent causative factor for omentin level decline, distinct from obesity [28,84,86,93,94]. The latest meta-analysis, however, has suggested that there are no significant changes in the circulating levels of omentin in non-obese PCOS individuals [99]. Regarding PCOS treatment, Tan et al. observed that administration of metformin may increase serum level omentin [86].

Interestingly, except for its highest expression in the visceral fat, omentin may be also found in the heart (as well as visceral pericardium), lungs, placenta and ovary [81,82,83,84]. The latter localization may suggest its further involvement in the pathogenesis of PCOS. Cloix et al. were the first to find a transcript of omentin in human granulosa-lutein cells (hGLCs), and found that its expression therein was two-fold higher in PCOS individuals, in comparison to control. Omentin detected in follicular fluid (FF) was also significantly higher than that in plasma in PCOS subjects, while no such relation pertained to individuals with infertility of non-endocrine origin [84]. These results were in agreement with the latest observations by Bongrani et al., who confirmed markedly elevated omentin concentrations in FF and granulosa cells, where both variables were positively correlated with BMI [100]. The exact role of omentin within the ovary requires further research; so far, Cloix et al. have concluded that omentin-1 may ameliorate IGF-1-induced steroidogenesis and insulin-like growth factor 1 receptor (IGF-1R) signaling through the induction of nicotinamide phosphoribosyltransferase (NAMPT) expression [84].

## 7. Gremlin

**Figure 5:**
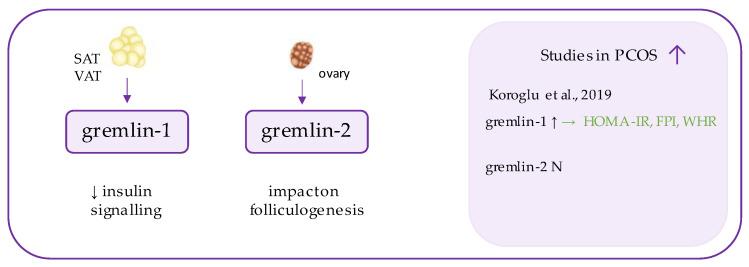
Role of gremlin in the pathogenesis of insulin resistance and PCOS. List of the studies assessing gremlins level in serum of the patients with PCOS. ↑/N indicates whether concentration level of gremlin was increased/unchanged in PCOS individuals (*p* < 0.05); → (green)—indicates positive correlation between serum omentin and particular indicators; HOMA-IR—Homeostatic Model Assessment for Insulin Resistance, FPI—fasting plasma insulin, WHR—waist-to-hip ratio.

Gremlins (Figure 5) are peptides belonging to DAN family, which function as extracellular antagonists of bone morphogenetic proteins (BMPs), especially BMP2 and BMP4 [101,102]. BMPs are highly conserved proteins, which exhibit transforming growth factor β (TGF-β) activity and regulate cell differentiation during embryogenesis and later stages of life [103]. Gremlins (1 and 2) neutralize those ligands by binding to them and preventing their interaction with receptors and signaling [102].

It has been shown that gremlin expression is high in the subcutaneous adipose tissue and, to a greater extent, in the visceral adipose tissue [104]. Hedjazifar et al. have recently shown that gremlin-1 may antagonize insulin signaling and reduce its glucose-mediated response. They detected increased transcription of gremlin mRNA in individuals with T2DM, glucose intolerance, non-alcoholic steatohepatitis (NASH), and non-alcoholic fatty liver disease (NAFLD) [104]. Furthermore, dysregulation of gremlin (characterized by its increased level together with elevated BMP4) has been observed in a population with hypertrophic obesity [105]. Hammarstedt et al. have concluded that, despite elevated synthesis of BMP2/4, the activity of morphogens may be impaired after binding to inhibitory gremlin [105].

There has only been one study assessing the serum concentration of gremlin in PCOS patients, to the best of our knowledge [29]. It presented significantly increased serum gremlin levels in the affected individuals, in comparison to the controls, and positive correlations with various markers of insulin resistance, such as insulin levels, HOMA-IR and waist-to-hip ratio (WHR) were observed. These observations related only to gremlin-1, while no significant results were provided for gremlin-2 [29]. This is consistent with the results presented by Hedjazifar et al., who focused on other conditions associated with insulin resistance [104]. We may conclude that deviations of circulating gremlin-1 in PCOS may reflect the metabolic disturbances in affected individuals. This places gremlin-1 as a potential biomarker of insulin resistance, and the aspect regarding the involvement of both gremlins in ovarian dysfunction remains promising in terms of future studies on PCOS pathogenesis.

Interestingly, even more research has been devoted towards involvement of gremlins in the regulation of late and (more recently) early stages of folliculogenesis [106,107,108]. BMPs have also been implicated as intra-ovarian regulators of follicle function and steroidogenesis, but the exact functions of their inhibitors (except for gremlin there are also noggin, chordin, and follistatin) is less well-established [109]. Glister et al. observed that gremlin antagonized BMP2- and, less potently, BMP4-induced suppression of androgen secretion but did not affect responses to BMP6 and BMP7 [109].

At the cellular level, Anti-Mullerian Hormone (AMH) acts similarly to BMP2/4, displaying TGF-β activity, and exerting an inhibitory effect on early follicular recruitment by preventing the entry of primordial follicles into the growing pool [110]. In the studies by Nilsson et al., it has been shown that both gremlins have the ability to reverse not only BMP4 activity, but also AMH in vitro [108]. Importantly, AMH is 2–4 times increased in subjects with PCOS in comparison to healthy females, thus having several implications for the pathogenesis of PCOS [110,111]. However, it was gremlin-2—not 1—that was expressed in primordial follicles, and Nilsson et al. concluded that gremlin-2 was part of the signaling network (together with multiple growth factors) that regulates the primordial-to-primary follicle transition [108]. Interestingly, in numerous studies on different conditions associated with ovarian dysfunction, it has been investigated that increased gremlin concentration may reflect defects in folliculogenesis [112,113,114,115].

## 8. Galectin-3

**Figure 6:**
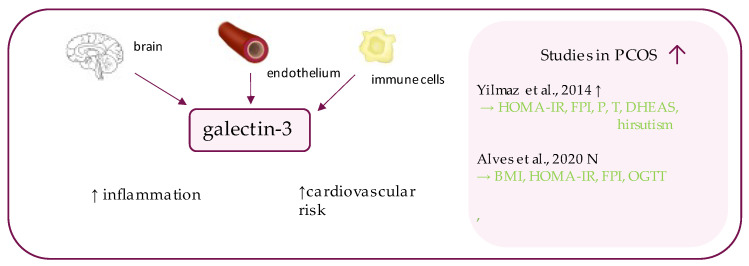
Role of galectin-3 in the pathogenesis of insulin resistance and PCOS. List of the studies assessing galectin-3 level in serum of the patients with PCOS. ↑/N indicates whether concentration level of galectin-3 was increased/ unchanged in PCOS individuals (*p* < 0.05); → (green)—indicates positive correlation between serum galectin-3 and particular indicators; HOMA-IR—Homeostatic Model Assessment for Insulin Resistance, FPI—fasting plasma insulin, P—progesterone, T—testosterone, DHEAS—dehydroepiandrosterone sulphate, BMI—body mass index, OGTT—oral glucose tolerance test.

Galectin-3 (Gal-3) (Figure 6), previously known as MAC-2 [116] or CBP-35 [117], is one of the fifteen members of the beta-galactoside-binding lectins family [118], which was discovered in the early 1970s [119]. This multi-functional, 35-kDA protein coded by the LGALS3 gene (located on chromosome 14), is expressed i.a. in the neural, immune and epithelial cells and is involved in the regulation of basic cellular processes, such as the cell cycle or cell growth [120,121]. As the most studied representative of the lectin family, Gal-3 has so far been associated with the pathogenesis of cancer, cardiovascular diseases, and arthritis, among others [121,122]. Gal-3 has recently been intensively investigated as a prospective biomarker of metabolic status in PCOS patients, as it has been shown to be related to insulin resistance, pre-diabetes and diabetes mellitus. Moreover, Gal-3 is also believed to exhibit pro-inflammatory properties [123,124,125]. In animal studies, the administration of Gal-3 contributed to glucose intolerance and insulin resistance, whereas the addition of its inhibitor reversed the effect [126]. 

Several studies have been conducted in order to assess serum Gal-3 levels and its relation to metabolic parameters in PCOS individuals. Alves et al. have performed a study on 44 women with PCOS and 25 healthy controls [127]. Although this study reported no significant differences between Gal-3 level in both groups, its concentration was positively associated with BMI, oral glucose tolerance test (OGTT), insulin level and HOMA-IR in affected individuals. The study contradicts the results obtained by Yilmaz et al., in which PCOS patients had statistically significantly higher Gal-3 levels than control individuals [30]. Furthermore, correlations were not only observed between serum Gal-3 levels and HOMA-IR, but also between Gal-3 and the levels of following hormones: insulin, progesterone, testosterone, and dehydroepiandrosterone sulphate [30]. The relation between Gal-3 level and hirsutism remains controversial, as contradictory results have been reported [30,128]. 

In the studies that divided PCOS patients into sub-groups, depending on whether or not individuals exhibited features associated with metabolic syndrome, significantly higher Gal-3 levels have been observed in subjects with metabolic syndrome [129]. Additionally, in this study, positive correlations were discovered between galectin and systolic and diastolic blood pressure, as well as with triglyceride levels [129]. Gal-3 level seem to be not only a possible marker of PCOS, but may also enable the identification patients at higher risk of developing metabolic or cardiovascular complications [129].

## 9. Neuregulin-4

**Figure 7:**
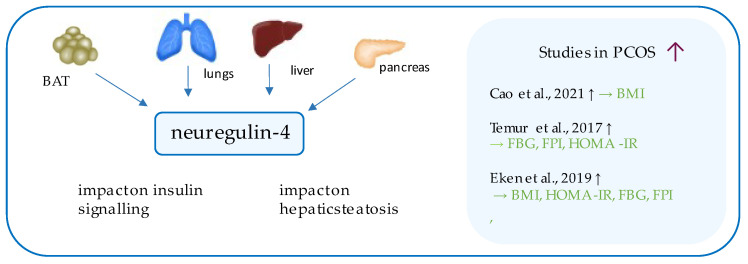
Role of neuregulin-4 in the pathogenesis of insulin resistance and PCOS. List of the studies assessing neuregulin-4 level in serum of the patients with PCOS. ↑ indicates that concentration level of neuregulin-4 was increased in PCOS individuals (*p* < 0.05); → (green)—indicates positive correlation between serum neuregulin-4 and particular indicators; BMI—body mass index; FBG—fasting blood glucose, FPI—fasting plasma insulin, HOMA-IR—Homeostatic Model Assessment for Insulin Resistance.

Neuregulin-4 (Figure 7) is a novel peptide, encoded in humans by the NRG4 gene located on chromosome 15, which is counted among the so-called batokines, as it is secreted mainly by brown adipose tissue (BAT) [130,131]. However, its expression was also detected in several internal organs [132]. Like the other members of this family, neuregulin-4 binds to representatives of ErbB/HER family receptors, which regulate cell–cell interactions through cell metabolism improvement, stimulation of cell proliferation, or inhibition of apoptosis [133,134].

Although numerous animal studies considering neuregulin-4 (NRG4) have been performed, there are limited data in humans. NRG4 is known to serve as a one of the factors improving insulin sensitivity [135], protecting the liver from fatty liver disease (FLD) [131] and its low circulating level may potentially serve as a risk factor for gestational diabetes mellitus [136]; however, the NRG4–PCOS relationship is poorly represented in the literature. 

In 2017, Temur et al. reported considerably higher serum levels of NRG4 in PCOS patients than in healthy controls [137]. High sensitivity C-reactive protein (Hs-CRP), low-density lipoproteins (LDL-C), HDL-C and hormones profile (LH; insulin; total testosterone; sex hormone binding globulin, SHBG; dehydroepiandrosterone sulfate, DHEA-SO_4_), as well as other metabolism-related factors such as fasting blood glucose (FBG), FAI, and HOMA-IR, were also elevated in comparison to healthy individuals. Positive correlations between NGR4 level and FBG, insulin, HOMA-IR and hs-CRP were observed in the study group. HOMA-IR, and hs-CRP proved to be independent markers associated with NRG4 [137]. Hypothetically, the high neuregulin level in PCOS subjects could be interpreted as a result of problems with NRG4 binding to epidermal growth factor receptor (EGFR); however, more research needs to be conducted to confirm this hypothesis [137].

As up to 60% of patients suffering from PCOS are obese [138], Eken et al. have deepened the research by comparing NRG4 levels in obese and non-obese PCOS patients, compared to obese and non-obese healthy controls [139]. Consistent results were obtained: the highest serum NRG4 levels were noted in obese PCOS patients, the second highest in non-obese PCOS, and the lowest in the obese and non-obese control groups. Additionally, circulating NRG4 was significantly associated with insulin resistance indicators, markers of obesity, and hormonal levels [139]. The most recent study investigating the impact of weight reduction on serum NRG4 levels in obese adolescent girls with PCOS [31] has also confirmed a higher initial NRG4 level in overweight patients [31]. In addition, the study showed that even a short (one-year-long) lifestyle intervention, including change of diet, increased physical activity and avoidance of sedentary behavior may enable to obtain NRG4 levels comparable to healthy control levels from the beginning of the study [31]. 

The aforementioned studies have emphasized the association of NRG4 with metabolic disorders: obesity seems to trigger secretion of NR4, and its high levels might be a form of adjustment to the low-grade chronic inflammation in PCOS; however, further research is required [31,137,139].

## 10. Xenopsin-Related Peptide

**Figure 8:**
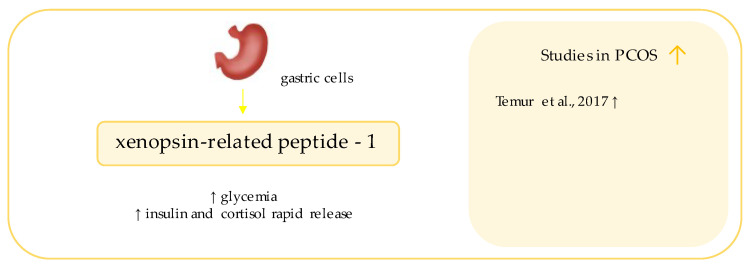
Role of xenopsin-related pepide in the pathogenesis of insulin resistance and PCOS. List of the studies assessing xenopsin-related peptide level in serum of the patients with PCOS. ↑ indicates that concentration level of neuregulin-4 was increased in PCOS individuals (*p* < 0.05).

Xenopsin-related peptide-1 (XP-1) (Figure 8) is a sister octapeptide to one found in the skin of *Xenopus laevis* [140]; which, in amphibians, may be a part of the defense system against predators [141].

So far, it has been detected, inter alia, in mammalian gastric mucosa cells (in humans and canines) [142] and in the gastric juice of humans diagnosed with duodenal ulcers [143]. Nevertheless, it remains a molecule with no well-established role. In the animal studies, the administration of artificially synthesized XP-1 to canines induced hyperglycemia and rapid glucagon and cortisol releases. A lesser, but still viable, effect was observed for gastrin and insulin [144,145].

Xenopsin-related peptide-1 had not gathered any attention as a subject of the gynecological endocrinology research until 2017, when Temur et al. considered XP-1 as a potential biomarker of insulin resistance in PCOS subjects [32]. In this study, XP-1 was found to be significantly higher in PCOS patients than in healthy controls [32]. Values of fasting serum insulin, C-reactive protein (CRP), and indicators of insulin resistance were higher in the affected individuals [32]. Interestingly, no correlations between XP-1 and lipid profile, insulin resistance indicators, or hormones were observed in the study group; however, XP-1 levels were significantly correlated with age, insulin, and HOMA-IR in the controls [32]. 

The insulin signaling pathway has been shown to be considerably disturbed in the course of PCOS [146]. XP-1 may be one of the markers predicting the risk of PCOS [32]. Although the results of the study seem to be promising, it must be noted that the study was limit by use of a small number of participants, what highlights the importance of conducting further research.

## 11. Xenin

**Figure 9:**
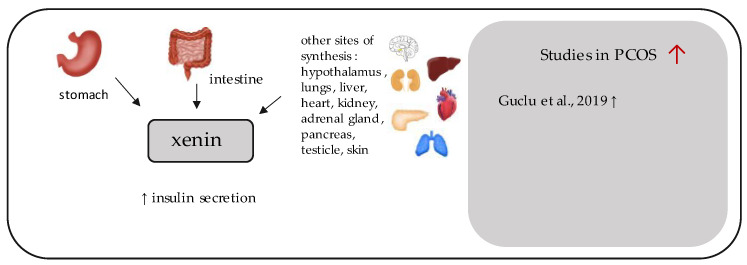
Role of xenin in the pathogenesis of insulin resistance and PCOS. List of the studies assessing xenin in serum of the patients with PCOS. ↑ indicates that concentration level of xenin was increased in PCOS individuals (*p* < 0.05).

Xenin (Figure 9) is a 25-amino acid peptide, which was initially extracted from the human gastric, duodenal, and jejunal mucosa by Feurle et al. in 1992 [147]. It was also detectable in human serum, it affected exocrine pancreatic secretion, and its concentration increased in the blood after meals [147]. Over the years, it has been shown that xenin may exhibit several other functions within the gastrointestinal tract, such as delaying gastric emptying, the regulation of gut motility and electrolyte transport, the contraction of the gallbladder, appetite suppression, and the inhibition of gastric acid production, and it may be also produced by other organs, such as hypothalamus, lung, liver, heart, kidney, adrenal gland, pancreas, testicles, and skin [148,149,150]. Its 6 C terminal amino acid sequence is identical with amphibian octapeptide xenopsin, as well as to a 13-amino acid peptide termed neurotensin [147,151]. Therefore, xenin may also bind to neurotensin receptor 1 (NTSR-1), but not NTSR-2 in the gut and evince similar biological activity [150,151]. Nonetheless, specific xenin receptors have not been identified [148,151]. 

It has recently been shown that xenin may amplify glucose-dependent insulinotropic peptide (GIP)-mediated insulin secretion [152]. Importantly, xenin is co-localized with GIP within K enteroendocrine cells in the small intestine [153]. Not only does exclusive xenin-25 exert such an incretin effect, but its smaller derivative fragment peptides also do, especially the C-terminal octapeptide fragment xenin-8 (xenin 18–25) [152,154,155,156]. There is currently a lot of research devoted to analogues of xenin-25 that are less vulnerable to degradation and that could be considered as new potential antidiabetic drugs (e.g., xenin-25-Gln, xenin-25[Lys13PAL], or [D-Ala2]GIP/xenin-8-Gln) [148], as their administration in animal studies has enhanced metabolic control and glucose homeostasis [157]. The rationale for such treatment may be provided by the blunted response to GIP-stimulated insulin release in humans with T2DM, its anorexigenic effect, and its possible impact on the reduction of lipogenesis and increase in lipolysis [152,158,159,160]. 

In terms of its association to PCOS, so far, only one study assessing the concentration of this peptide has been conducted [33]. It demonstrated that xenin-25 levels were significantly increased in a small group of 31 women with PCOS compared to the control group of 30 healthy, regularly menstruating women. The optimal cutoff value of xenin-25 for predicting PCOS was 32.60 pg/mL, giving a sensitivity of 61.3% and a specificity of 86.7%. The groups did not differ from each other regarding markers of insulin sensitivity [33].

## 12. Neudesin

**Figure 10:**
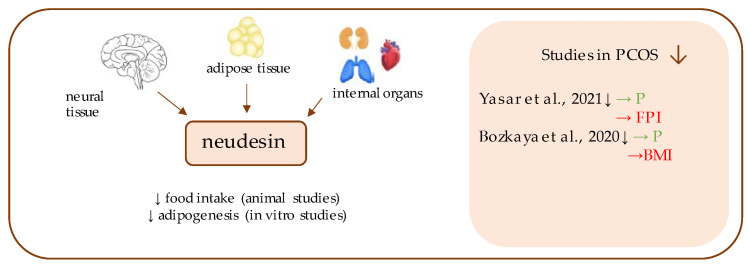
Role of neudesin in the pathogenesis of insulin resistance and PCOS. List of the studies assessing neudesin in serum of the patients with PCOS. ↓ indicates that concentration level of neudesin was decreased in PCOS individuals (*p* < 0.05); → (green)—indicates positive correlation between serum neudesin and particular indicators; → (red)—indicates negative correlation between serum neudesin and particular indicator; P—progesterone; FPI—fasting plasma insulin; BMI—body mass index.

Neudesin (Figure 10), also known as a GIG47 oncogene or the neuron-derived neurotrophic factor (NENF) [161,162], is a 171-amino acids protein with neurotrophic activity that belongs to the membrane-associated progesterone receptor (MAPR) family [163]. It was initially found in embryonic and puerperal mouse tissue and, soon after, its expression was also confirmed in humans. Human neudesin (containing 172 amino acids) preserves 91% sequential similarity with its mouse homolog [164]. So far, research has mostly focused on its synthesis within the neural tissue [164,165]; however, neudesin is also expressed in the adipose tissue [166], in the internal organs (e.g., lungs, heart, and kidneys) and in the numerous neoplasms [167].

Although neudesin was identified less than 20 years ago [164], it still remains mostly unexplored, and is only now becoming a subject of metabolism-related studies (in diseases, relating to obesity, diabetes mellitus, insulin resistance and PCOS) [34,167,168,169,170,171,172]. 

The study by Bozkaya et al. comprised the first attempt to investigate a putative link between PCOS diagnosis and serum neudesin concentration [168]. The authors revealed its lowered level in PCOS patients, and an inverse association between neudesin levels and PCOS risk was determined [168]. Moreover, while progesterone levels are known to be decreased in the course of PCOS [173], in the study by Bozkaya et al., its level did not significantly differ between the study and control groups, and a positive correlation between neudesin and progesterone was noted in the affected individuals [168]. An insignificant difference in the neudesin level was observed between PCOS group with and without insulin resistance [168]. Although higher insulin, HOMA-IR, FPG, and FPI values were noted in PCOS subjects, no link was proved in the statistical analysis between neudesin and insulin resistance-related parameters [168]. 

Similarly, in the newest study by Yasar et al., the neudesin level was found to be lowered in the PCOS group, and was positively correlated with progesterone and insulin levels [34]. 

Therefore, it is highly possible that serum neudesin variations are related to the pathophysiology of PCOS, however, it is not known whether it is one of the triggers, or a consequence of the disorder [34].

## 13. Lipocalin-2

**Figure 11:**
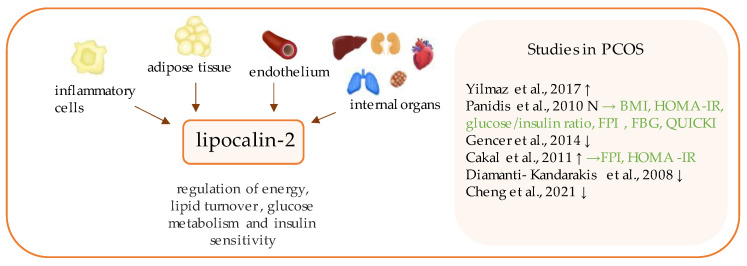
Role of lipocalin-2 in the pathogenesis of insulin resistance and PCOS. List of the studies assessing lipocalin-2 in serum of the patients with PCOS. ↑/↓/N indicates whether concentration level of lipocalin-2 was increased/decreased/unchanged in PCOS individuals (*p* < 0.05); → (green)—indicates positive correlation between serum lipocalin-2 and particular indicators; BMI—body mass index; HOMA-IR—Homeostatic Model Assessment—Insulin Resistance; FPI—fasting plasma insulin; FBG—fasting blood glucose; QUICKI—quantitative insulin sensitivity check index.

Lipocalin-2 (LCN2) (Figure 11), also identified as 24p3, siderocalin, or neutrophil gelatinase-associated lipocalin (NGAL), is the representative of the lipocalins family [174,175]. This 178-amino acid protein occurs in three different forms: as a 25-kDa monomer, a 45-kDa homodimer and a 135-kDa heterodimer, forming a complex structure with matrix metalloproteinase 9 (MMP-9) [176]. Lipocalin-2 expression has been detected in several types of cells, including neutrophils, adipocytes, macrophages, endothelial cells, endometrial cells, splenic cells and hepatocytes [177,178]. LCN-2 is known to regulate inflammatory pathways and cytokine secretion and it plays a role in chronic inflammatory diseases, such as obesity, TDM2, or NASH [174,179]. It has been confirmed, in animal studies that LCN2 deficiency may protect from the development of insulin resistance through regulation of lipoxygenase and cachexin levels in adipose tissue [180]. Since the first decade of the 21^st^ century, LCN2 has gathered a growing interest, due to its potential role as a biomarker for PCOS; nevertheless, the results of studies aiming to confirm this hypothesis remain ambiguous [35,177,181,182,183,184,185,186].

Cakal et al. have performed a cross-sectional study investigating LCN2 as an insulin resistance marker in the PCOS population and found considerably higher serum lipocain-2 levels in PCOS individuals than in age- and BMI-matched healthy controls [185]. Similar outcomes have been presented by Yilmaz et al. [35]. In the study by Panidis et al., LCN2 serum levels were slightly elevated, but the results were not statistically significant, despite the greater number of participants. However, the study and control groups did not differ in terms of HOMA-IR [177]. Indeed, HOMA-IR values significantly differed between the PCOS and control groups in the formerly mentioned studies by Cakal et al. [185] and Yilmaz et al. [35].

Surprisingly, there have also been reports of lower LCN2 levels in PCOS patients than in healthy individuals [181,186,187], thus emphasizing the need for larger studies. There also exist divergent data when it comes to relation of LCN level to HOMA-IR, as a correlation between them has been determined in some studies [177,185], but not found in others [35,186]. In the latest study, Cheng et al. observed the association between LCN-2 serum level and diabetes mellitus progression in PCOS patients [187].

In terms of obesity, Martínez-García et al. investigated higher lipocalin-2 in non-obese PCOS patients compared with non-obese controls. Conversely, in obese PCOS subjects, the serum lipocalin-2 level was lower than that in the obese controls [183]. Indeed, some researchers have concluded that elevated LCN2 levels are linked to the obesity associated with PCOS, not to PCOS alone [177,184]. Therefore, further studies are required.

## 14. Discussion

There exists a variety of new proteins/peptides that may participate in the process of glucose and insulin homeostasis. In our article, we focused only on selected ones—nesfatin-1, preptin, myonectin, omentin-1, gremlin-1, galectin-3, neuregulin-4, xenopsin-related peptide, xenin, neudesin, and lipocalin-2—and investigated their associations with insulin resistance in patients with PCOS. Most research so far has focused on the study of adipokines—agents synthesized in the adipose tissue [6,188,189]. However, as we have shown in our manuscript, peptides potentially involved in induction of insulin resistance may be synthesized in other organs, for example, in the pancreas (e.g., preptin), hypothalamus (e.g., nesfatin), skeletal muscles (e.g., myonectin), intestine (e.g., xenin), immune cells (e.g., lipocalin-2) and, in terms of adipose tissue, it can occur either in the visceral one (e.g., omentin, gremlin-1) or brown adipose tissue (e.g., neuregulin-4). Taken together, this demonstrates that the process of energy turnover is intricate and involves many organs. 

The studies discussed in our paper were mostly focused on examining the relationships between PCOS and several peptides, which may be considered as markers of insulin resistance. This topic has recently attracted attention in the scientific world, and many studies in that field were conducted, not only in relation to PCOS, but also in respect to other conditions involving defective insulin signaling, such as obesity, diabetes mellitus, and gestational diabetes [190,191,192,193].

When it comes to the markers we have reviewed, significant variations in their concentrations were observed in the sera of individuals with PCOS. Basing our conclusion on the most predominant tendency determined in the studies, we may speculate that the concentrations of the markers preptin, gremlin-1, galectin-3, neuregulin-4, xenin-25, xenopsin related peptide in serum may be elevated, while those of nesfatin-1, myonectin, omentin-1, neudesin may be decreased in PCOS individuals. Nevertheless, there also exist studies presenting contradictory results in terms of the levels of these markers (e.g., in some studies, its concentration is increased, while in others, decreased or unchanged), as well as when considering the correlation between the marker concentration levels and those of insulin resistance indicators. Most conflicting data exist for lipocalin-2. 

Possible explanations for such inconsistencies may include differences in study design, observation performed on too small groups, various criteria for selection of case and control groups (e.g., regarding BMI and insulin resistance indicators), and the administration of differing kits for the measurement of marker levels.

In future research, we recommend taking into consideration whether the variation in a marker’s concentration is related to presence of PCOS itself, or whether it is a result of concomitant obesity and/or insulin resistance. Assessment of marker concentration levels after meal/glucose administration may also be valuable. Other interesting approaches may include analysis of markers’ concentrations before and after introducing treatment for PCOS, examination of a relationships between the peptides and intestinal dysbiosis, and the roles of age and ethnicity in affecting marker concentrations.

The strength of our study is taking into consideration protein candidates for IR biomarkers, which have not been extensively reviewed previously. We summarized and updated recent progress in this field, increasing the general knowledge about PCOS pathogenesis and the new peptides involved in the metabolic homeostasis and energy turnover. Beside simple description of the results from the studies, we also highlighted a few important controversial issues that were encountered during our literature search. Moreover, whenever the data were available, we provided information related not only to the pathogenesis of insulin resistance, but also to the impact of selected peptides on ovarian function. The main limitation of the paper is the fact that the manuscript was designed in a form of a narrative review. However, we put a lot of effort into identification of studies conducted on females with PCOS, and we believe that the majority of them were discussed. Moreover, some of the selected peptides are considered quite novel and, sometimes, an insufficient number of studies had been performed to analyze the concentration levels of the particular markers. Although the results of the studies often appear to be promising, there is an urgent need for further research in the field. Therefore, some of our assumptions may be considered as preliminary hypotheses, which require further clarification through future research.

## 15. Conclusions

In this paper, we focused on less commonly known peptides and proteins that may potentially interact with insulin production and signaling through insulin receptor. We reviewed studies assessing the roles of these agents in the pathogenesis of PCOS. Based on our analysis, we may provide the conclusion that, for the markers preptin, gremlin-1, galectin-3, neuregulin-4, xenin-25, xenopsin-related peptide, the serum concentration was increased, while it was decreased for nesfatin-1, myonectin, omentin-1, neudesin. Nevertheless, it must be acknowledged that there also exist studies presenting contrary results, where the most conflicting data exist for lipocalin-2. Therefore, the debate is still open and further research is required to establish the roles of these markers more adequately, along with their involvement in pathogenesis of PCOS and the possibility and utility of measuring their levels in clinical practice. 

## Figures and Tables

**Table 1:** Recent studies comparing serum fasting levels of each of the markers between patients with PCOS and healthy controls.

Marker	Author	PCOS Patients (*n*)	Control Patients (*n*)	Level of the Marker (PCOS) ^1^	Level of the Marker (Control) ^1^	Insulin Resistance Indicators (*p*-Value) ^2^
Nesfatin-1	Demir Çaltekin et al., 2021 [25]	44	40	17.08 ± 13.8 ng/mL (*p* < 0.001)	36.8 ± 20.7 ng/mL	FPG (*p* = 0.999); FPI (*p* < 0.001); HOMA-IR (*p* = 0.001); VAI (*p* = 0.144)
Preptin	Mierzwicka et al., 2018 [26]	73	61	8.88 ± 3.89 ng/mL (*p* = 0.0255)	7.53 ± 2.53 ng/mL	FPG (*p* = 0.244); FPI (*p* = 0.0076); OGTT (*p* = 0.0665); HOMA-IR (*p* = 0.0009); QUICKI (*p* = 0.00834); Matsuda Index (*p* = 0.0006); 120 min Ins (*p* = 0.0007)
Myonectin	Demir & Guler 2020 [27]	72	72	6.77 ± 1.96 ng/mL (*p* < 0.001)	9.14 ± 2.87 ng/mL	FPG (*p* = 0.044); FPI (*p* < 0.001) OGTT (*p* = 0.218); HOMA-IR (*p* < 0.0001); HbA1c (*p* = 0.265)
Omentin	Franik et al., 2020 [28]	86	72	210.5 ng/mL (149–302.7) (*p* < 0.001)	515.9 ng/mL (256.3–779.0)	FPG (*p* < 0.001); FPI (*p* < 0.01); HOMA-IR (*p* < 0.01)
Gremlin-1	Koroglu et al., 2019 [29]	50	30	1.89 ng/mL (1.36–2.57) (*p* = 0.001)	1.36 ng/mL (0.64–1.92)	FPG (*p* = 0.190); FPI (*p* = 0.056); HOMA-IR (*p* = 0.000)
Galectin-3	Yilmaz et al., 2014 [30]	56	41	3588.77 ± 1566.94 ng/dL (*p* < 0.001)	2491.33 ± 812.04 ng/dL	FBG (*p* = 0.144); HOMA (*p* = 0.508)
Neuregulin-4	Cao &Hu, 2021 [31]	52	43	8.12 ± 3.03 ng/mL (*p* = 0.031)	4.22 ± 1.25 ng/mL	Serum C peptide (*p* = 0.012); FPI (*p* = 0.026)
Xenopsin-related peptide	Temur et al., 2017 [32]	40	38	6.49 ± 1.57 ng/mL (*p* = 0.001)	5.29 ±1.45 ng/mL	FBG (*p* = 0.134); FPI (*p* = 0.002); HOMA-IR (*p* = 0.003)
Xenin-25	Guclu et al., 2019 [33]	31	30	220.79 ± 259.4 pg/mL (*p* = 0.007)	68.58 ± 152.78 pg/mL	FPG (*p* = 0.437); FPI (*p* = 0.345); HOMA-IR (*p* = 0.478)
Neudesin	Yasar et al., 2021 [34]	180	100	1.19 ± 1.08 ng/mL (*p* = 0.015)	2.12 ± 1.04 ng/mL	FBG (*p* = 0.170); FPI (*p* = 0.004); HOMA-IR (*p* = 0.004); HbA1C (*p* = 0.231)
Lipocalin-2	Yilmaz et al., 2017 [35]	44	47	55.74 ± 17.54 ng/mL (*p* < 0.011)	36.46 ± 19.62 ng/mL	FBG (NS); FPI (*p* = 0.02); HOMA-IR (*p* = 0.014)

^1^ Data expressed (if available) as mean ± SD or median (interquartile range); ^2^ each bracket contains *p*-value (*p*) comparing difference between serum marker’s concentration of PCOS and control patients; n-number of the patients in the study, PCOS—polycystic ovarian syndrome; FPG—fasting blood glucose, FPI—fasting plasma insulin, HOMA-IR—Homeostatic Model Assessment—Insulin Resistance, VAI—visceral adiposity index; OGTT—oral glucose tolerance test; QUICKI—Quantitative Insulin Sensitivity Check Index; 120 min Ins—insulin serum level measured after 120 min in the oral glucose tolerance test; Hb1Ac—Hemoglobin A1C; NS—non-significant.

## Data Availability

Not applicable.
